# Dynamic changes in lactate levels within the first 24 hours in septic patients as a prognostic indicator: A retrospective cohort study utilizing latent class growth analysis

**DOI:** 10.17305/bb.2023.9259

**Published:** 2023-12-01

**Authors:** Shifeng Li, Tao You, Meili Liu, Yan Hao, Xinyue Li, Zhiyang Wang, Fang Huang, Jun Wang

**Affiliations:** 1 Department of Intensive Care Medicine, The First Affiliated Hospital of Soochow University, Suzhou, Jiangsu, China; 2 Department of Hematopathology, The First Affiliated Hospital of Soochow University, Suzhou, Jiangsu, China

**Keywords:** Latent class growth analysis (LCGA), the Medical Information Mart for Intensive Care-IV database (MIMIC-IV), lactate, intensive care unit (ICU), mortality

## Abstract

Elevated lactate levels are common in sepsis patients. This study aimed to assess the effect of dynamic changes in lactate levels within the first 24 h following admission on patient prognosis. We extracted data from the Medical Information Mart for Intensive Care (MIMIC)-IV database and classified patients using latent class growth analysis (LCGA). This analysis classified sepsis patients into different groups based on dynamic changes in lactate levels during the initial 24 h post-admission, dividing this time frame into four periods (0–3 h, 3–6 h, 6–12 h, and 12–24 h). The highest lactate level recorded in each period was then used for patient classification. We subsequently compared the baseline characteristics and outcomes between these different groups. Our study encompassed 7830 patients, whom LCGA successfully divided into two classes: class 1 (steady lactate class) and class 2 (increasing lactate class). Class 2 demonstrated a worse clinical status at baseline, as indicated by vital signs, disease severity scores, and laboratory results. Importantly, class 2 also had a significantly higher 28-day mortality rate than class 1 (55.6% vs 13.5%, *P* < 0.001). In conclusion, LCGA effectively categorized sepsis patients into two distinct groups based on their dynamic changes in lactate levels during the first 24 h post-admission. This methodology has potential utility in clinical practice for managing sepsis patients.

## Introduction

Lactate is a product of anaerobic glycolysis, and under normal circumstances, production and consumption of lactate are relatively balanced [1, 2]. A change in blood lactate levels often indicates an imbalance in the body’s oxygen supply [3]. Early studies by Weil and Afifi and other researchers led to the use of blood lactate concentration as an indicator of tissue dysfunction and the necessity of resuscitation in critically ill patients [4–6]. Lactate is currently used as an indicator of insufficient tissue oxygenation and as a marker of the need for resuscitation in shock [7, 8]. Given that many studies have shown that increased blood lactate levels are associated with a higher mortality rate in sepsis patients, serum lactate is often used to assess the severity of sepsis, monitor response to treatment, and predict prognosis of sepsis patients [9–11]. The recent Sepsis-3 guidelines recommend that a persistent serum lactate level greater than 2 mmol/L should be used as a standard for the clinical definition of septic shock, even when adequate fluid resuscitation is provided [12].

In clinical practice, however, the level of blood lactate is typically considered a simple static observation, and little is known about the relationship between the dynamics of serum lactate and the severity of sepsis. Although some researchers have proposed the concept of lactate clearance to evaluate these dynamics, there is controversy regarding the use of these measurements [13–15].

Therefore, our general purpose was to develop a method for assessing the trends of lactate levels over time in sepsis patients. Latent class growth analysis (LCGA) is a statistical method that can distinguish similar subpopulations (classes) within a large heterogeneous population. LCGA can be used for fitting and analyzing continuous data and has been applied in previous studies for clinical data analysis [16, 17]. At the same time, some studies have also proved the value of LCGA classification in predicting prognosis [18]. We used LCGA to identify different classes of sepsis patients based on the dynamic changes in lactate levels during the first 24 h after admission and then compared the baseline characteristics and outcomes of patients in the different classes.

## Materials and methods

### Database

The data were extracted from the Medical Information Mart for Intensive Care (MIMIC)-IV database [19], which was released on March 16, 2021 [20]. All of the de-identified data were from patients who were diagnosed with sepsis and were treated in a critical care unit of the Beth Israel Deaconess Medical Center from 2008 to 2019. Patients provided consent when the data were collected. After passing an examination and obtaining a certification, researchers could access this database.

### Data extraction

The data were extracted using Navicat Premium version 15.0.12, and they included demographic parameters, vital signs, medical history, disease severity scores, laboratory results, length of intensive care unit (ICU) stay, and lactate levels measured during four different periods of time after the ICU admission (0–3 h, 3–6 h, 6–12 h, and 12–24 h).

### Study design

**Figure 1.**
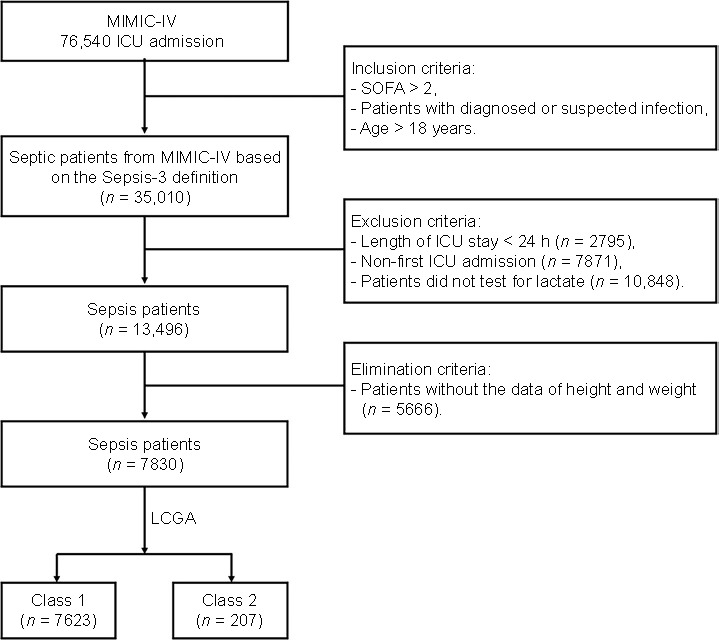
**Flowchart of the study**. It respresents the disposition of sepsis patients from the MIMIC-IV database (*n* ═ 76,540), and the LCGA of patients into class 1 (steady lactate, *n* ═ 7623) and class 2 (increasing lactate, *n* ═ 207). MIMIC-IV: Medical Information Mart for Intensive Care-IV database; LCGA: Latent class growth analysis; SOFA: Sequential Organ Failure Assessment score; ICU: Intensive care unit.

The records of 76,540 patients were initially examined (Figure 1). The inclusion criteria were a diagnosis of sepsis according to the Sepsis-3 criteria [12] and an age older than 18 years. The exclusion criteria include the length of ICU stay shorter than 24 h, previous admissions to the ICU, and patients who have not undergone lactate testing. The elimination criteria involve patients for whom the data on height and weight are unavailable.

### Statistical analysis

The expectation maximization (EM) interpolation method was used to infer missing values. This method assumes that the missing data were missing at random, using the existing data and a specified model to formulate the most accurate estimate [21]. This constitutes a maximum-likelihood (ML) approach [22], which is suitable for the analysis of continuous variables. Our intention was to use this method to interpolate the missing lactate data. According to statistics, the missing lactate data were as follows: The number of missing lactate at 3 h entries is 2580 (32.9%); the number of missing lactate at 6 h entries is 4530 (57.8%); the number of missing lactate at 12 h entries is 3956 (50.5%); and the number of missing lactate at 24 h entries is 3935 (50.2%). Through the application of interpolation, we were able to obtain complete datasets and proceeded with the next step of the analysis.

The LCGA was used to identify patient subpopulations (classes) that had different dynamic changes in serum lactate levels during the 24 h after ICU admission so that classes with different trajectories could be compared [23, 24]. This method used lactate levels measured during the four different periods within the first 24 h after admission (see above). Then, the highest measured lactate level during each period was used to classify patients into different groups, and the baseline characteristics and outcomes of the different groups were compared. R version 4.0.3 and MedCalc Software (Ostend, Belgium) were used for the LCGA analysis. The Akaike Information Criterion (AIC) and the Bayesian Information Criterion (BIC) can be used as the fitting indices, with smaller values indicating a better fit. Entropy is used to assess the accuracy of classification, and a value closer to 1 signifies better classification accuracy. Guided by AIC and BIC values [25, 26], we identified three target classes. We used entropy to evaluate the accuracy of these classification results [27]. Finally, a visual trajectory map was used to provide a clinical interpretation of the different classes. The primary outcome measure was mortality within 28 days after admission, and this outcome was compared between the different classes. The significance of differences in the baseline characteristics of patients among the different classes was determined using a *t*-test. These characteristics included sex, age, underlying diseases, hospitalization days, Sequential Organ Failure Assessment (SOFA) score, Acute Physiology Score (APS III), use of a vasoactive drug, use of mechanical ventilation, use of continuous renal replacement therapy (CRRT), and related laboratory data that reflect organ function and prognosis.

## Results

### Latent class growth analysis

**Figure 2.**
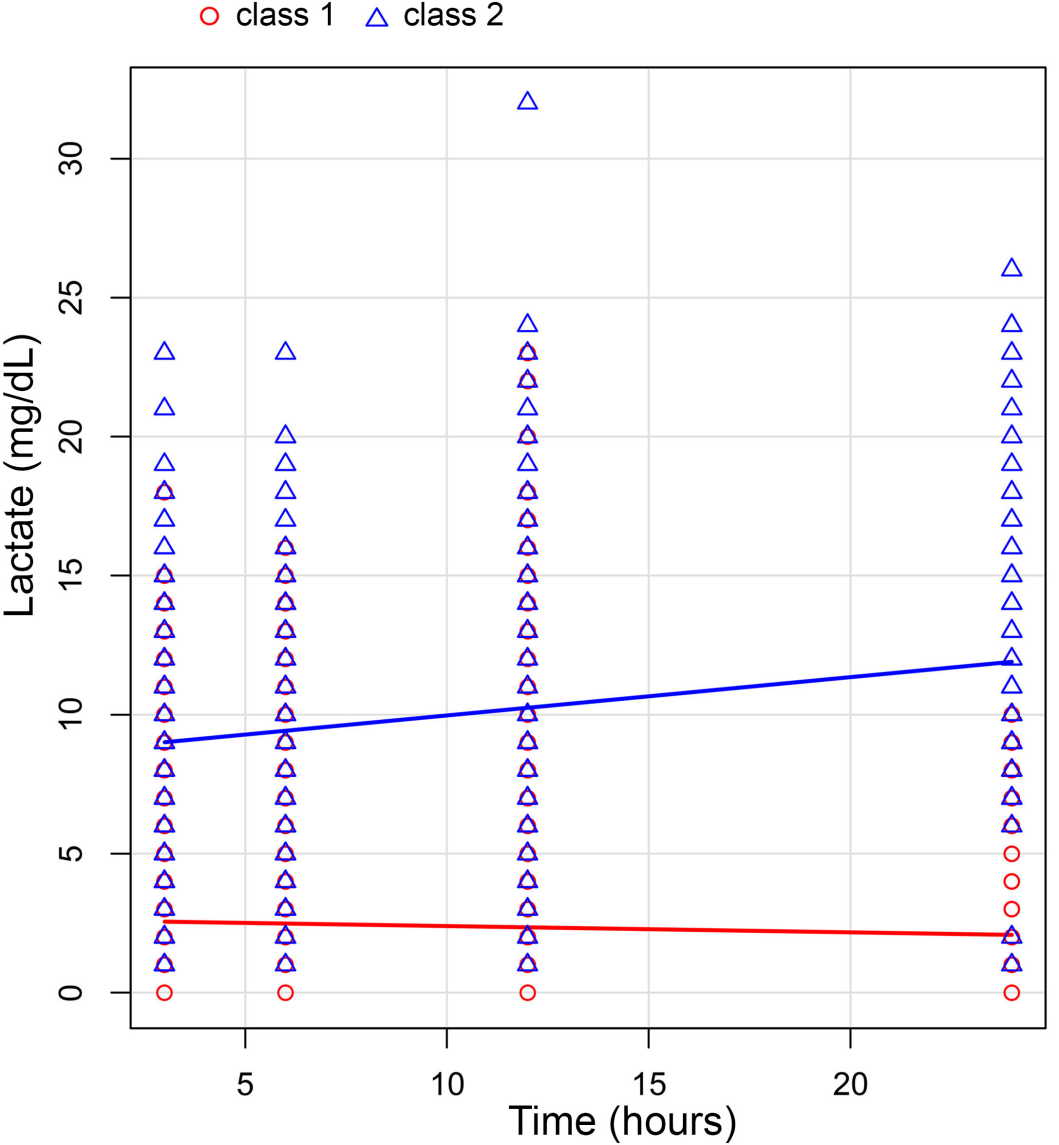
**Changes of lactate levels (mg/dL) from admission to 24 h in class 1 (steady lactate class, red circles) and class 2 (increasing lactate class, blue triangles) after grouping using the LCGA.** Lines indicate linear regressions for each class. LCGA: Latent class growth analysis.

**Figure 3.**
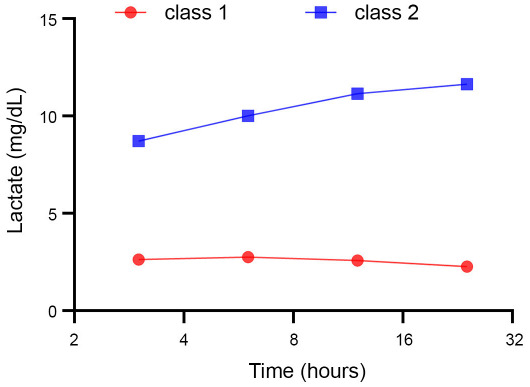
**The lactate values in each time period and their changing trends after the LCGA.** Following the LCGA, the lactate values for each time period are represented by dots, allowing the visualization of their changing trends for both classes. The lactate values of class 1 were low, exhibiting a flat trend, while the lactate values of class 2 were higher, displaying a noticeable upward trend. LCGA: Latent class growth analysis.

After the application of the inclusion and exclusion criteria and the elimination of patients lacking data on height, weight, and admission date, we used LCGA to analyze 7830 sepsis patients according to dynamic changes in lactate levels during the 24 h after the ICU admission (Figure 1). The LCGA results established different classes of patients based on the values of AIC, BIC, and entropy (Table 1). Following the division into three classes, both the AIC and BIC values exhibited significant decreases, indicating that the analysis was reliable. However, since the first class comprised zero patients, we decided to proceed with two classes based on the dynamic changes observed in lactate levels (Figure 2). We defined “class 1” (*n* ═ 7623) as the steady lactate class and “class 2” (*n* ═ 207) as the increasing lactate class. After the LCGA, we discovered that the lactate value of class 1 was low with a flat trend, while the lactate value of class 2 was higher, showing a certain upward trend (Figure 3).

**Table 1:** Measures of statistical fit and classification accuracy for models with classes 1, 2, or 3

	**Classes**	**AIC**	**BIC**	**SSA-BIC**	**Entropy**	**Class 1**	**Class 2**	**Class 3**
Model 1	1	121961.37	122003.17	121984.10	1	100	NA	NA
Model 2	2	121967.37	122030.06	122001.46	<0.001	97.62	2.37	NA
Model 3	3	117619.95	117703.544	117665.41	0.82	0.00	97.35	2.64

AIC: Akaike Information Criterion; BIC: Bayesian Information Criterion; SSA-BIC: Sample-size-adjusted Bayesian Information Criterion.

**Table 2:** Baseline characteristics and outcomes of class 1 (steady lactate) and class 2 (increasing lactate) based on LCGA

	**Class 1 (*n* ═ 7623)**	**Class 2 (*n* ═ 207)**	*P*
*Demographics*			
Age, years	67.22 (56.70–76.83)	65.34 (56.90–74.96)	0.181
Male sex	4737 (62.1)	126 (60.9)	0.765
Height, cm	170.00 (163.00–178.00)	173.00 (161.50–178.00)	0.656
Weight, kg	80.60 (68.00–96.00)	80.00 (67.75–95.70)	0.908
*Vital signs*			
Heart rate	84.80 (76.65–96.69)	95.67 (84.19–107.14)	**<0.001**
MAP, mmHg	75.00 (70.20–80.80)	71.11 (65.66–75.88)	**<0.001**
*Medical history*			
Hypertension	2093 (27.5)	40 (19.3)	0.012
Diabetes	641 (21.5)	33 (15.9)	0.065
COPD	721 (9.5)	21 (10.1)	0.832
CKD	1899 (24.9)	60 (29.0)	0.210
*Disease severity at admission*			
SOFA score	3.00 (2.00–5.00)	6.00 (4.00–9.00)	**<0.001**
APS III score	52.00 (36.00–75.00)	97.00 (81.00–120.50)	**<0.001**
SAPS II	39.00 (31.00–49.00)	59.00 (44.00–69.00)	**<0.001**
*Laboratory results*			
WBC, ×10^9^/L	15.00 (11.00–19.90)	17.85 (11.93–26.02)	**<0.001**
Platelets, ×10^9^/L	197.00 (146.00–265.00)	167.00 (110.25–239.25)	**<0.001**
BUN, mg/dL	22.00 (15.00–37.00)	34.50 (23.00–53.25)	**<0.001**
Creatinine, mg/dL	1.10 (0.80–1.80)	2.30 (1.60–3.70)	**<0.001**
ALT, IU/L	32.00 (18.00–81.00)	196.00 (45.00–752.00)	**<0.001**
NT-pro BNP, pg/mL	4383.00 (1229.00–12629.00)	5353.00 (1443.00–12291.00)	0.647
Troponin-T, ng/mL	0.13 (0.05–0.49)	0.30 (0.09–1.01)	**<0.001**
Bilirubin, mg/dL	0.70 (0.40–1.50)	1.90 (1.00–4.40)	**<0.001**
*Treatment*			
Vasoactive drugs	853 (11.2)	53 (25.6)	**<0.001**
Invasive mechanical ventilation	3859 (50.6)	139 (67.1)	**<0.001**
CRRT	200 (2.6)	40 (19.3)	**<0.001**
*Outcome*			
ICU stay, days	3.56 (1.94–7.38)	4.06 (1.75–9.79)	0.254
Hospital stay, days	9.48 (5.79–16.64)	9.58 (2.31–19.98)	0.014
Death at 28 days	1026 (13.5)	115 (55.6)	**<0.001**

Data are presented as median (IQR) or *n* (%). The bolded *P* values represent those that showed statistical significance. LCGA: Latent class growth analysis; MAP: Mean arterial pressure; COPD: Chronic obstructive pulmonary disease; CKD: Chronic kidney disease; SOFA: Sequential Organ Failure Assessment score; APS III: Acute Physiology Score III; SAPS II: Simplified Acute Physiology Score II; WBC: White blood cells; BUN: Blood urea nitrogen; ALT: Alanine transaminase; NT-pro BNP: N-terminal pro-hormone brain natriuretic peptide; CRRT: Continuous renal replacement therapy; ICU: Intensive care unit.

### Baseline characteristics of different classes

After the LCGA, we compared the baseline characteristics of the two classes (Table 2). There were no significant differences in demographic data (age, sex ratio, height, and weight). However, the increasing lactate class had a higher mean heart rate (95.67 vs 84.80 bpm, *P* < 0.001) and a lower mean arterial pressure (71.11 vs 75.00 mmHg, *P* < 0.001). The steady lactate class had a higher prevalence of hypertension (27.5% vs 19.3%, *P* ═ 0.012), but the classes did not differ in their prevalence of chronic kidney disease (CKD), chronic obstructive pulmonary disease (COPD), or diabetes. The increasing lactate class had higher disease severity scores (SOFA, APS III, and Simplified Acute Physiology Score II [SAPS II], all *P* < 0.001) and higher levels of all laboratory indicators (all *P* < 0.001) except N-terminal pro-hormone brain natriuretic peptide (NT-pro BNP). An analysis of treatment measures indicated that the increasing lactate class had a greater use of vasoactive drugs (25.6% vs 11.2%, *P* < 0.001), CRRT (19.3% vs 2.6%, *P* < 0.001), and invasive mechanical ventilation (67.1% vs 50.6%, *P* < 0.001). The analysis of the three outcome measures indicated that the two classes did not differ in days of hospitalization or days in the ICU, but the 28-day mortality was higher in the increasing lactate class (52.1% vs 13.5%, *P* < 0.001).

**Figure 4.**
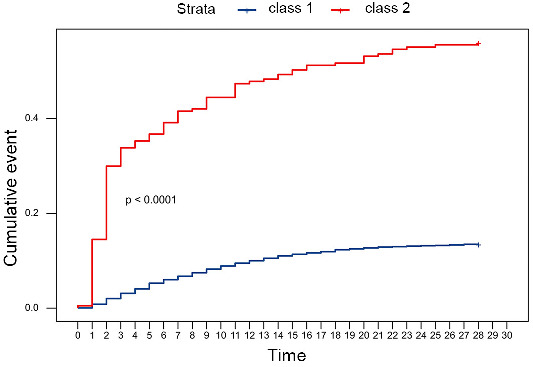
**The survival curve analysis of class 1 (steady lactate class, blue) and class 2 (increasing lactate class, red).** The survival curve analysis showed that the 28-day mortality rate was significantly higher in the increasing lactate class (log-rank test: *P* < 0.0001).

In alignment with the aforementioned results, the survival curve analysis further showed that the 28-day mortality rate was significantly higher in the increasing lactate class (log-rank test: *P* < 0.0001, Figure 4).

## Discussion

Lactate is produced in the brain, skeletal muscles, intestines, and many other organs, and is often thought of as a product of glycolysis [28, 29]. However, according to the latest research, lactate also plays an important physiological role in tumor immunity by inhibiting the cytotoxicity of immune cells and promoting the immune escape of tumor cells [30–32]. Many studies have examined the relationship between serum lactate levels and disease severity and prognosis of sepsis patients [33–35]. The purpose of this study was to use LCGA to examine the relationship between the dynamic changes in lactate levels during the first 24 h after admission and the prognosis of sepsis patients.

We used lactate measurements from four different time periods during the 24 h after the ICU admission (0–3 h, 3–6 h, 6–12 h, and 12–24 h). The highest value measured in each period was extracted from the MIMIC-IV database, and missing values were interpolated using EM. The LCGA method categorized our large population of sepsis patients into two classes. This classification was based on the dynamic changes in lactate levels observed during the first 24 h after the ICU admission. Specifically, class 1 consisted of patients who exhibited no increase in lactate levels, while class 2 encompassed those patients who experienced an increase in these levels. Our comparisons of the baseline characteristics of patients in these two classes indicated that patients in the increasing lactate class presented with more severe disease manifestations than those in the steady lactate class.

Lactate is an important prognostic biomarker in patients with sepsis. Previous research reported that the lactate level at admission had a significant positive correlation with poor prognosis in patients with sepsis [36]. However, the timing of lactate measurement during the 24 h after admission can affect its prognostic value [37]. It is difficult to measure the lactate level at precise predefined times for patients admitted to an ICU, and a single measurement is often used. When the static evaluation of the relationship between lactate levels at admission and prognosis is blocked, dynamic changes in lactate levels within a fixed period of time can be used to predict prognosis. The measurement of lactate clearance is an indicator of dynamic changes in lactate levels [38], but lactate clearance is not a suitable metric when the lactate levels are increasing. We used LCGA to classify sepsis patients based on the dynamic changes in lactate levels observed during the first 24 h following admission. This statistical method is increasingly used for data analysis in diverse disciplines [27]. The advantage of using LCGA is that it does not require simultaneous lactate measurements in all patients; instead, it considers the dynamic changes in lactate levels, identifying them as either increasing or decreasing. This makes LCGA a highly suitable technique for analyzing lactate measurements performed during the first 24 h after admission.

We successfully used LCGA to classify sepsis patients based on dynamic changes in lactate levels. Notably, we also found that the increasing lactate class had a significantly higher lactate level than the steady lactate class. This is consistent with previous studies which reported a correlation between increasing initial lactate level and poor prognosis in sepsis patients [39]. Our comparison of the baseline characteristics between the two classes showed that the disease severity scores at admission were worse in the increasing lactate class, indicating that patients in the increasing lactate class had a more advanced and serious form of the disease. Our baseline laboratory indicators analysis also showed that the increasing lactate class had higher levels of white blood cells (WBCs), platelets, blood urea nitrogen (BUN), creatinine, alanine transaminase (ALT), and bilirubin, further confirming that the patients in the increasing lactate class were suffering from more severe infections and exhibited poorer organ function [40, 41]. Our analysis of outcomes indicated that the two classes had no significant differences in hospital stay or ICU stay, but the increasing lactate class had an increased 28-day mortality rate. Our survival curve analysis confirmed that there was a higher 28-day mortality rate in the increasing lactate class (*P* < 0.0001). Interestingly, we found that the two classes had no significant differences in laboratory indicators of cardiac function (NT-pro BNP). This observation may suggest that there isn’t a clear connection between lactate level and cardiac function, but this topic requires further study.

There were several limitations to this study. Firstly, our analysis was based on a public database and we did not use our own data to verify the results. Secondly, our conclusions were based on a retrospective analysis, a type of study that is associated with several types of biases. Notably, the database was incomplete, and despite employing multiple interpolation to compensate for this, we could not entirely avoid the bias caused by missing data. Our use of LCGA method with data on dynamic changes in lactate levels during the first 24 h after admission of sepsis patients indicated that patients in the increasing lactate class had a more severe baseline disease and poorer prognosis at 28 days.

Importantly, these lactate data were all collected within 24 h after ICU admission, meaning that the approach described here provides results very soon after admission. This conclusion suggests that, in clinical practice, we may need to give more attention to class 2 patients and that implementing more proactive measures may improve these patients’ prognoses.

## Conclusion

We used the LCGA method to divide sepsis patients into two classes, based on the dynamic changes in lactate levels during the first 24 h after admission. Patients in the increasing lactate class experienced more severe organ dysfunction, required more invasive treatments, and had worse prognoses. Our results suggest that measuring dynamic changes in lactate levels during the first 24 h after admission of sepsis patients may be useful for the selection of more appropriate interventions, although this needs to be further verified through more multicenter prospective clinical studies.
